# Flavonoids mitigate neurodegeneration in aged *Caenorhabditis elegans* by mitochondrial uncoupling

**DOI:** 10.1002/fsn3.1956

**Published:** 2020-10-23

**Authors:** Injeong Cho, Hyun‐Ok Song, Jeong Hoon Cho

**Affiliations:** ^1^ Department of Biology Education College of Education Chosun University Gwangju Korea; ^2^ Department of Infection Biology Wonkwang University School of Medicine Iksan Jeonbuk Korea

**Keywords:** *C. elegans*, flavonoids, mitochondrial uncoupling, mitophagy, neurodegeneration

## Abstract

Dietary supplementation of flavonoids has been shown to reduce the severity of neurodegenerative disorders such as dementia, Parkinson's disease, and Alzheimer's disease by their antioxidant effects. However, their low bioavailability*in vivo* raises the question of how much their antioxidant capacity actually contributes to the mitigating effects. The physicochemical properties of flavonoids suggest they could function as mitochondrial uncouplers. Moreover, mitochondrial uncoupling alleviated neurodegeneration in *Caenorhabditis elegans* during aging in previous research. Therefore, we investigated whether various flavonoids (fisetin, quercetin, apigenin, chrysin, catechin, and naringenin) could reduce neuronal defects by mitochondrial uncoupling in *C. elegans*. Both neuronal defects and mitochondrial membrane potential were reduced in aged worms in nearly all of the flavonoid treatments suggesting that flavonoids may reduce neurodegeneration in *C. elegans*. However, there was no significant reduction of neuronal defects in mitophagy‐deficient *pink‐1/pdr‐1* double mutants under flavonoid treatments. These results suggest that flavonoids could function as mitochondrial uncouplers to mitigate neurodegeneration in aged *C. elegans*, possibly via a PINK1/Parkin mitophagy process.

## Graphical abstract

Flavonoid treatment reduced neurodegeneration in aged *C. elegans* in association with reduced mitochondrial membrane potential, indicated by TMRE staining. The results were reproduced in uncoupling mutant, *ucp‐4*, and chemical uncoupler DNP treated worms, but not in *pink‐1;pdr‐1* mutant worms. Therefore, flavonoids could function as a mitochondrial uncoupler to mitigate neurodegeneration in aged *C. elegans*, possibly via a PINK1/Parkin mitophagy process.

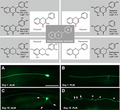

## 1 INTRODUCTION

The increased incidence of aging‐associated diseases has initiated intensive research in this field (Prince et al., 2013). In particular, many studies have focused on understanding the mechanisms underlying the development of neurodegenerative diseases, including dementia, Parkinson's disease, Alzheimer's disease, and Huntington's disease. Several studies have shown that neurodegenerative diseases are associated with an accumulation of dysfunctional mitochondria in neuronal cells as well as other cells (Bossy‐Wetzel et al., 2008; Fang et al., 2019; Kerr et al., 2017; Villace et al., 2017). Grimm and Eckert found that unhealthy mitochondria are associated with increased levels of reactive oxygen species (ROS) and ATP deficits (Grimm & Eckert, 2017). Excessive levels of ROS can damage mitochondrial lipids, proteins, and DNAs, subsequently affecting mitochondrial function (Harper et al., 2004; Hu & Liu, 2011). For the neuronal cell to survive and function properly, it is essential to maintain a healthy mitochondrial population (Misgeld & Schwarz, 2017; Rose et al., 2017).

Mitophagy, an autophagy process for removing damaged or excessive mitochondria, is one of the mitochondrial quality‐control processes (Ashrafi & Schwarz, 2013). Mitophagy is triggered by changes in mitochondrial membrane potential (Ψ_m_). PTEN‐induced putative kinase 1 (PINK1) protein in mitochondria senses mitochondrial membrane depolarization, and Parkin amplifies this signal to initiate mitophagy (Jin et al., 2010; Kondapalli et al., 2012). Mutations in the genes encoding these proteins lead to development of neurodegenerative diseases (Narendra et al., 2008, 2010). Moreover, chemical uncouplers, such as 2,4‐dinitrophenol (DNP) and carbonyl cyanide m‐chlorophenyl hydrazone (CCCP), also induce mitophagy by reducing the mitochondrial membrane potential (Liu et al., 2015; Narendra et al., 2010). In addition, mitochondrial uncoupling by DNP mitigates neuronal defects via mitophagy in aged *Caenorhabditis elegans* (Cho et al., 2016).

Flavonoids are a class of secondary metabolites found largely in fruits and vegetables. These low‐molecular weight phenolic compounds have long been used as dietary supplements to improve human health due to their beneficial antioxidant, anti‐inflammatory, and anticarcinogenic properties (Havsteen, 2002). Recent research has demonstrated that polyphenol compounds stimulate brain activity and mitochondrial function (Naoi et al., 2019). Further, flavonoids may protect neuronal cells from some of the adverse effects of aging‐associated diseases and neurodegenerative diseases (Spencer, 2007; Youdim & Joseph, 2001). Regular intake of flavonoid‐rich foods showed decreased risk of dementia, Alzheimer's disease, and Parkinson's disease (Commenges et al., 2000; Gao et al., 2012; Shishtar et al., 2020). The antioxidant activity of flavonoids is considered to be a prominent mechanism in reducing the severity of aging‐associated diseases since many of the neurological disorders, diabetes, and vascular defects can be exacerbated or caused by free radicals (Pandey & Rizvi, 2009). Mitochondria are one of major contributors of intracellular ROS owing to mitochondrial respiration (Boveris, 1984; Boveris & Chance, 1973; Boveris et al., 1972). While some mechanisms of flavonoids' antioxidant effects are reasonably well understood, the mechanism underlying the role of flavonoids in moderating the effects of aging‐associated diseases is still unclear. The cellular concentration of flavonoids in the brain or in blood is less than 1 µM, which seems unlikely to be sufficient to scavenge ROS effectively; by comparison, vitamin C requires a concentration in the mM range for antioxidant activity (Halliwell, 2008; Manach & Donovan, 2004; Rechner et al., 2002; Sandoval‐Acuna et al., 2014; Williamson & Manach, 2005). Furthermore, due to their physicochemical properties, flavonoids can act as protonophores and thereby dissipate mitochondrial membrane potentials (Stevens et al., 2018). Therefore, it is worth investigating whether flavonoids mitigate aging‐associated neurodegeneration via mitochondrial uncoupling and thereby inducing mitophagy.

In the present study, we used *C. elegans* as the model organism to study neuronal function. The ease of neuronal manipulation and observation in *C. elegans* makes it an excellent tool for research, and its rapid life cycle is well suited to aging‐related studies. To our knowledge, no study so far has reported an uncoupling role for flavonoids in aging‐associated neurodegeneration in *C. elegans*. Hence, we investigated whether flavonoids reduce neurodegeneration in aged *C. elegans* by mitochondrial uncoupling and triggering mitophagy.

## 2 MATERIALS AND METHODS

### 2.1 Strains and constructs

The strains, Bristol N2 wild‐type, *ucp‐4* deletion mutant (*ok195*, CY121), *pdr‐1* (*gk448*, VC1024), and *zdIs5* ([p*_mec‐4_*GFP], CZ10175), were provided by the *Caenorhabditis* Genetics Center (CGC) at the University of Minnesota. *pink‐1* deletion mutant (*tm1779*) was provided by the Mitani laboratory through the National Bio‐Resource Project (Japan). For observation of mechanosensory neurons, all strains were crossed with *zdIs5*. The *ucp‐4* mutant was crossed with *zdIs5* to obtain *zdIs5;ucp‐4*. The *pink‐1* mutant was crossed with *zdIs5* to obtain *zdIs5;pink‐1*, which was further crossed with *pdr‐1* to obtain *pink‐1;pdr‐1* double mutants.

The culture conditions were in accordance with the standard protocols (Brenner, 1974). For reliable worm maintenance, freeze‐dried OP50 from LabTIE (LabTIE B. V., Rosmalen, The Netherlands) was used according to the manufacturer's instructions. To achieve synchronous populations of *C. elegans* and prevent internal egg hatching, fluorodeoxyuridine (FUdR) was applied to the top of the NGM‐OP50 plates at a final concentration of 50 µM. Worms at L4 stage were transferred to the FUdR‐treated plates.

### 2.2 Observation of neuronal defects

Neuronal defects were determined by observation of anterior lateral microtubule (ALM) and posterior lateral microtubule (PLM) morphological abnormalities, specifically an outgrowth in ALM cells, and/or wavy, neuronal sprouts and branching in PLM cells (Chen et al., 2013; Pan et al., 2011; Tank et al., 2011; Toth et al., 2012). When an ALM or PLM neuron exhibited any of the morphological abnormalities, the neuron was counted as one defect. All observations were performed using a fluorescence microscope (80i‐DS‐Fi1, Nikon). Chi‐squared tests were performed to compare DMSO‐treated control and flavonoid‐treated worms. *p*‐values < .05 were considered statistically significant.

### 2.3 DNP and flavonoid treatment

A stock solution (10 mM) of DNP (Sigma‐Aldrich, Saint Louis, MO, USA) in dimethyl sulfoxide (DMSO, Sigma‐Aldrich) was added to NGM medium at the desired final concentration before pouring into plates. The flavonoids fisetin, quercetin, apigenin, chrysin, catechin, and naringenin, were obtained from Cayman Chemical Company (Cayman Chemical Company, Ann Arbor, MI, USA). Flavonoid‐NGM plates were prepared using the same procedure mentioned for DNP. Worms at L4 stage were transferred to flavonoid‐containing NGM plates.

### 2.4 Tetramethylrhodamine ethyl ester assay

Tetramethylrhodamine ethyl ester (TMRE, Molecular Probes) is used as an indicator of mitochondrial membrane potential (Ψ_m_) owing to its cell permeability and cationic fluorescence (Ehrenberg et al., 1988; Farkas et al., 1989). TMRE in DMSO (50 µM) was applied to flavonoids‐NGM plates at a final concentration of 0.1 µM. After worms were exposed to flavonoid‐plates at the L1 stage, the worms were transferred to the TMRE plates at the L4 stage and incubated at 20°C for 4 hr. L4‐stage worms were used due to the intense staining of embryos at the adult stage. After staining, the worms were washed with M9 buffer and mounted on a slide (Yoneda et al., 2004). A fluorescence microscope was used for observation and imaging of the worms. Image J was used for the TMRE data analysis (Rasband, https://imagej.nih.gov/ij/). One‐way ANOVA analysis was performed, followed by an unpaired *t* test, for DMSO treatment and flavonoid treatments in worms.

## 3 RESULTS AND DISCUSSION

### 3.1 Physicochemical properties of flavonoids suggest a role as uncouplers

**Figure 1:**
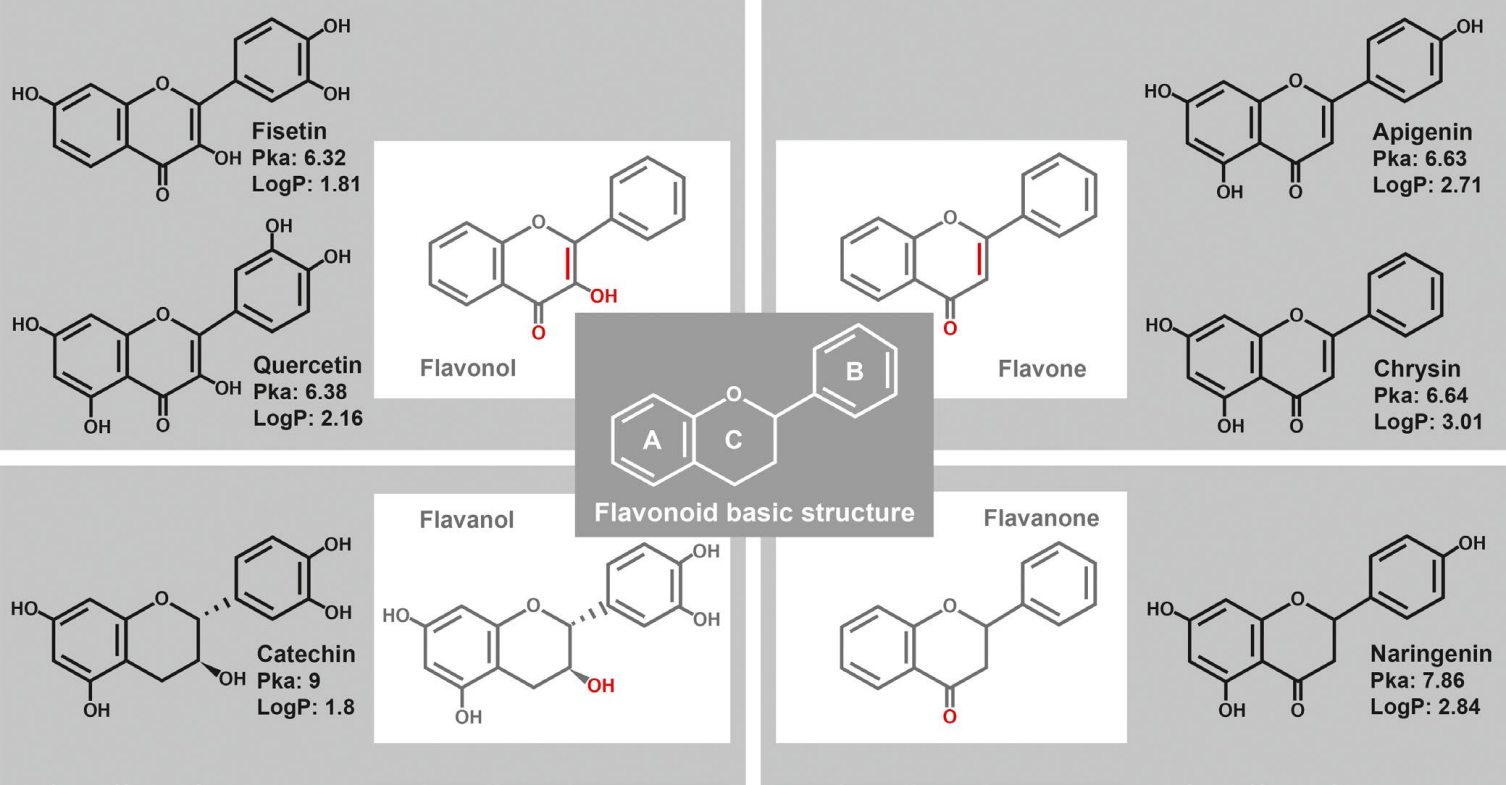
Chemical structure of flavonoids. The four subgroups of flavonoids and their pKa (acid dissociation constant) and LogP values are presented

Many studies have reported that the majority of flavonoids are potent antioxidants (Panche et al., 2016). The strong antioxidant properties of flavonoids can be attributed to their structure, which can donate electrons and stabilize the oxidized form of flavonoids (Koch et al., 2014). However, the physicochemical properties of flavonoids also favor their potential to act as protonophores due to their favorable pKa (acid dissociation constant, between 6 and 9) and moderate lipophilicity (LogP < 5) (Wishart et al., 2006, Figure 1). Therefore, we hypothesized that flavonoids can dissipate mitochondrial membrane potential (Ψ_m_) by functioning as mitochondrial uncouplers. Flavonoids are classified into four subclasses depending on the degree of unsaturation and oxidation of the C ring: flavonols, flavones, flavanols, and flavanones (Figure 1). We exposed *C. elegans* to at least one flavonoid from each category in order to determine the contribution of the protonophore‐like structure to flavonoid‐mediated mitochondrial uncoupling and prevention of neurodegeneration in aged *C. elegans*.

### 3.2 Flavonoids alleviate ALM and PLM neurodegeneration in aged *C. elegans*

**Figure 2:**
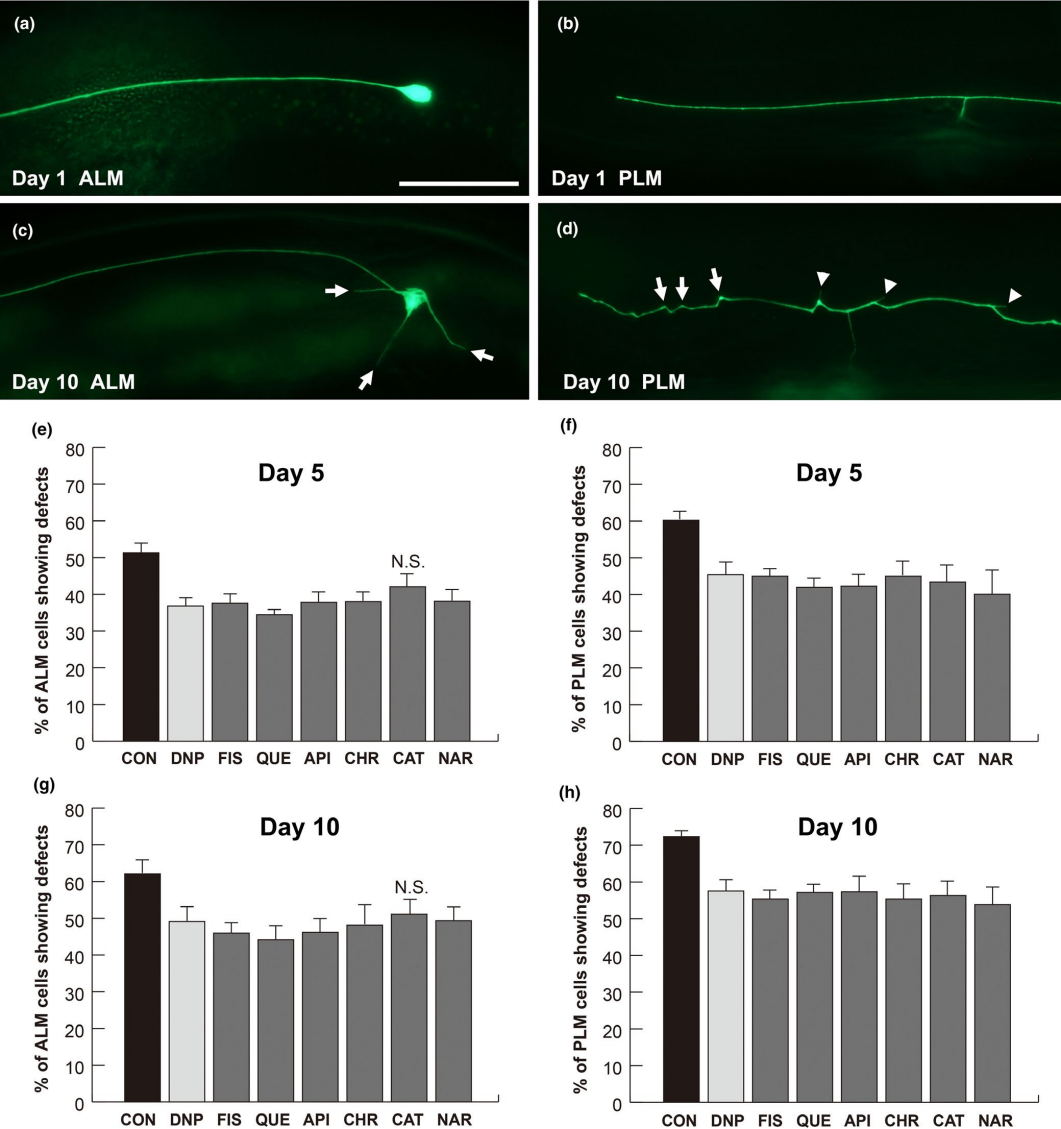
Flavonoids alleviate neurodegeneration in aged *C. elegans*. (a–d) Representative images of mechanosensory neurons in *zdIs5* [p*_mec‐4_*GFP] worms on day 1 (a and b) and day 10 (c and d). Arrows indicate outgrowth in ALM (c). Arrows and arrowheads indicate wavy processes and neuronal sprouting in the PLM, respectively (d). Scale bar = 50 μm. (e–h) ALM and PLM neuronal defects are presented as a percentage of the total ALM‐ and PLM‐scored neurons in worms treated with different flavonoids on day 5 (e and f) and day 10 (g and h). DNP, 2,4‐dinitrophenol; FIS, fisetin; QUE, quercetin; API, apigenin; CHR, chrysin; CAT, catechin; NAR, naringenin; ALM, anterior lateral microtubule; PLM, posterior lateral microtubule. Data are represented as means of 3 independent experiments ± *SD*. *n* > 120 worms per line in each experiment. Chi‐squared tests were performed to compare flavonoids treatment and DMSO treatment. *p*‐values are less than 0.05 unless specified otherwise (Table S1 for *p*‐values). N.S.; not significant

Previous studies have shown that the morphologies of mechanosensory neurons, ALM and PLM, were affected by aging in *C. elegans*. In addition, abnormalities such as soma outgrowth, wavy processing, and branching were easily observed and scored under microscope (Figure 2c and d) (Cho et al., 2016, 2017). Therefore, scoring of ALM and PLM neuronal defects was performed in reference worms (*zdIs5* [p_*mec‐4*_GFP]) to investigate the effects of flavonoids on aging‐associated neurodegeneration. According to Koch et al., flavonoid concentrations in the range of 0.1–200 µM resulted in increase the mean life span in *C. elegans* (Koch et al., 2014). Therefore, the concentrations 5, 10, 20, and 100 µM were used for feeding wild‐type N2 worms (L4 stage), followed by neuronal observation under a fluorescence microscope at days 1–15 after treatment (data not shown). No significant difference was observed in the number of neuronal defects between worms treated with 10 µM flavonoids and worms treated with 100 µM flavonoids. Based on these results, a 10 µM concentration of each flavonoid was used in the subsequent study to minimize any side effects. Since many of the aged worms die between day 12 and day 18, we decided to observe and monitor aging‐associated neurodegeneration at day 5 and day 10 in the flavonoid‐exposed worms.

It has been established that DNP reduces neuronal defects in aged worms; therefore, DNP was used as the positive control (Cho et al., 2016, 2017). As anticipated, both ALM and PLM defects in the worms treated with 10 µM DNP were lower than those in the DMSO controls at day 5 (29% and 24% reduction in ALM and PLM defects, respectively, Figure 2e and f) and at day 10 (21% in ALM and 21% in PLM, Figure 2g and h). Fisetin and quercetin, which are in the same flavonol category, both significantly decreased neuronal defects at days 5 and 10 (Figure 2e–h, Table S1 for *p*‐values). Quercetin‐treated worms showed 32% less ALM and 31% less PLM neuronal defects at day 5, and 30% less ALM and 21% less PLM neuronal defects at day 10 as compared to the DMSO‐treated worms (Figure 2e–h). Worms treated with fisetin exhibited decreased ALM and PLM neuronal defects as compared to the DMSO control at day 5 (28% and 25%, respectively, Figure 2e and f) and at day 10 (26% and 24%, respectively, Figure 2g and h).

The flavons, apigenin and chrysin, decreased neuronal defects compared to the DMSO‐treated control (Figure 2e–h, Table S1 for *p*‐values). The ALM neuronal defect was 26% less in apigenin‐treated worms than in the DMSO control worms at days 5 and 10 (Figure 2e and g). Apigenin‐treated worms showed decreased PLM defects at day 5 and at day 10 (30% and 21%, respectively, Figure 2f and h). The ALM neuronal defects in chrysin‐treated worms were reduced by 26% both at days 5 and 10 (Figure 2e and g), and the PLM defects were decreased by 23% both at days 5 and 10 (Figure 2f and h).

The flavanol catechin decreased PLM defects by 28% at day 5 and by 22% at day 10, respectively (Figure 2f and h), as compared to DMSO‐treated controls. However, ALM defects in catechin‐treated worms were not significantly different from that in control worms at both days 5 and 10 (Figure 2e and g). The flavanone, naringenin, decreased ALM and PLM defects by 25% and 34% at day 5, and by 21% and 25% at day 10, respectively (Figure 2e–h), as compared to DMSO‐treated controls.

### 3.3 Flavonoids alleviate neurodegeneration via mild mitochondrial uncoupling

**Figure 3:**
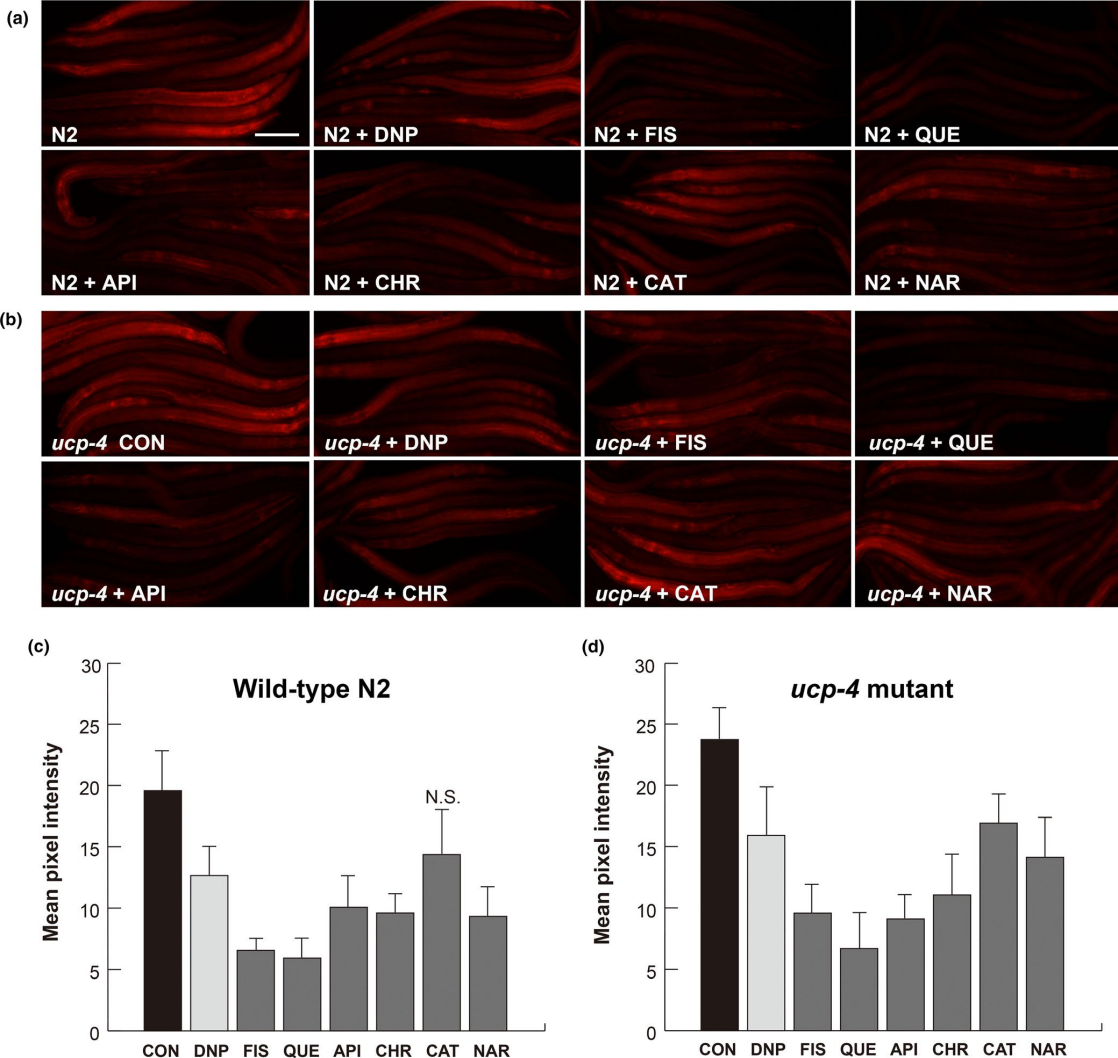
Flavonoids lower mitochondrial membrane potential in aged *C. elegans*. Representative images of TMRE staining (a and b). (a) N2 wild‐type worms treated with 100 μM DNP and 100 µM of FIS, QUE, API, CHR, CAT, and NAR. (b) *ucp‐4* mutant worms treated with 100 μM DNP and 100 µM of FIS, QUE, API, CHR, CAT, and NAR. Scale bar = 100 μm. (c and d) TMRE intensities in N2 worms (c) and *ucp‐4* mutant (d). Pixels in fluorescence images were quantified by Image J (Rasband, https://imagej.nih.gov/ij/). Data are represented as means of 3 independent experiments ± *SD*. *n* > 12 worms per line in each experiment. One‐way ANOVA analysis and *t* tests. *p* < .05; significant (Table S2 for *p*‐values). N.S.; not significant. DNP, 2,4‐dinitrophenol; FIS, fisetin; QUE, quercetin; API, apigenin; CHR, chrysin; CAT, catechin; NAR, naringenin; TMRE, tetramethylrhodamine ethyl ester

To investigate whether flavonoid‐mediated alleviation of neurodegeneration involves mitochondrial uncoupling, the mitochondrial membrane potential indicator TMRE was used (Yoneda et al., 2004). In preliminary experiments, TMRE staining was performed in worms treated with 10–100 µM flavonoids, and it was found that the 100 µM concentration was the most effective to visualize the differential TMRE‐staining pattern among tested groups (data not shown). Since we already confirmed that there was no substantial differences in neuronal defects between the 10 and 100 µM flavonoid treatments, 100 µM concentration of flavonoids was used to monitor mitochondrial membrane potential by TMRE staining. The worms were treated with 100 µM flavonoids from L1 stage and collected at L4 stage for TMRE staining. DNP, one of chemical uncouplers, was used as the positive control. Figure 3 represents TMRE‐stained worms at the L4 stage (Figure 3a and b, Table S2 for *p*‐values). The intensity of staining was less in worms treated with 100 µM DNP than that of the DMSO‐treated controls, which is consistent with our previous study (Cho et al., 2017). The intensity of staining in worms treated with fisetin and quercetin was markedly lower than that in control worms (67% and 70% reduction respectively; Figure 3a and c). Worms treated with apigenin and chrysin also exhibited a decrease in the intensity of staining as compared to control worms (49% and 51%, respectively, Figure 3a and c). The intensity of the staining in naringenin‐treated worms showed difference as compared to the control worms (53%, Figure 3a and c). However, the intensity of the staining in catechin‐treated worms showed slight decrease as compared to the control worms (Figure 3a and c). Except catechin, all tested flavonoids resulted in significantly reduced TMRE staining even compared to the positive control, DNP. These results suggest that flavonoids depolarize mitochondrial membrane potential.

For further confirmation, TMRE staining was performed in DMSO‐treated and flavonoid‐treated uncoupling‐defective *ucp‐4* mutants. *ucp‐4* mutants showed the highest TMRE intensity due to defective mitochondrial uncoupling (Figure 3b and d), and the intensity was decreased by treatment with 100 µM DNP (Figure 3b and d, Table S2 for *p*‐values). These results are consistent with the results of our previous study (Cho et al., 2016, 2017). Quercetin substantially reduced TMRE‐staining intensity in *ucp‐4* mutants, followed by fisetin (72% and 60% reduction, respectively; Figure 3b and d). Apigenin, chrysin, and naringenin also decreased TMRE‐staining intensity of *ucp‐4* mutants (Figure 3b and d). The least reduction in staining intensity was observed in catechin‐treated *ucp‐4* mutants (Figure 3b and d).

Regardless of flavonoid treatment, the intensity of TMRE fluorescence in aged worms was lower than that observed in L4 stage (data not shown). The low TMRE intensity in aged worms could be due to reduced TMRE uptake and accumulation, since TMRE accumulation in mitochondria depends on mitochondrial activity (Ehrenberg et al., 1988; Loew et al., 1993; Nicholls & Ward, 2000). Despite this limitation, however, TMRE is considered a practical tool for monitoring mitochondrial membrane potential in live cells and organisms (Farkas et al., 1989).

In TMRE staining, the largest intensity reduction (and consistent with neuronal defects results) was obtained for quercetin‐treated N2 and *ucp‐4* mutant worms (Figure 3a–d). Quercetin has been reported to act as an uncoupler to dissipate mitochondrial membrane potentials in isolated mitochondria and in whole H9c2 cells (Ravanel, 1986; Zholobenko et al., 2017). Moreover, quercetin inhibits NADPH oxidase that comprises mitochondrial complex I and subsequently lowers ROS production (Lagoa et al., 2011). Taken together, the reduction in TMRE staining by quercetin could be the combined result of direct uncoupling as a protonophore and indirect uncoupling by inhibition of mitochondrial complex I.

Recent research showed that catechin also inhibits mitochondrial complex I, resulting in reduced mitochondrial membrane potential and less hydrogen peroxide production in isolated mitochondria (Iglesias et al., 2019). In contrast to the results obtained from quercetin treatment, catechin‐treated N2 exhibited a decrease in TMRE‐staining intensity with minimal reduction in neuronal defects in this study. It has been reported that quercetin accumulates inside the mitochondria of Jurkat cells and is readily available (Fiorani et al., 2010). Therefore, the minimal effects of catechin may be attributed to its low bioavailability.

**Figure 4:**
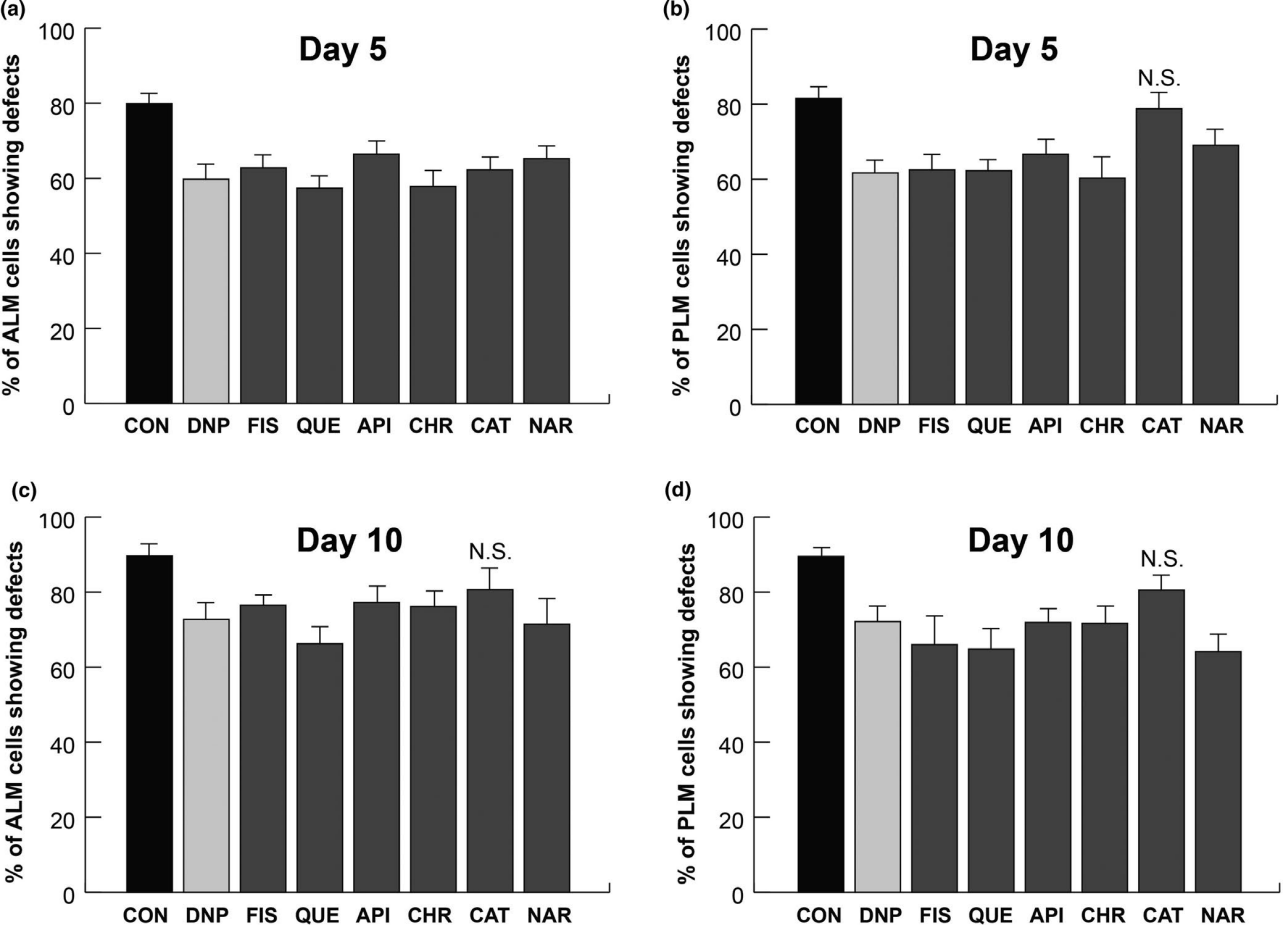
Flavonoids alleviate neurodegeneration in aged *ucp‐4* mutants. (a–d) ALM and PLM neuronal defects are presented as a percentage of the total ALM‐ and PLM‐scored neurons in DMSO‐treated and flavonoid‐treated *ucp‐4* mutants on day 5 (a and b) and day 10 (c and d). DNP, 2,4‐dinitrophenol; FIS, fisetin; QUE, quercetin; API, apigenin; CHR, chrysin; CAT, catechin; NAR, naringenin; ALM, anterior lateral microtubule; PLM, posterior lateral microtubule. Data are represented as means of 3 independent experiments ± *SD*. *n* > 120 worms per line in each experiment. Chi‐squared tests were performed to compare flavonoids treatment and DMSO treatment. *p*‐values are less than .05 unless specified otherwise (Table S3 for *p*‐values). N.S.; not significant

To investigate the relationship between TMRE intensity and neurodegeneration in *ucp‐4* mutants, neuronal observation experiments were performed in *ucp‐4*;*zdIs5* worms. The *ucp‐4* mutants were treated with 10 µM flavonoids or 10 µM DNP, and neuronal defects were scored at day 5 and day 10 post‐treatment. DNP‐treated *ucp‐4* mutants exhibited 25% less ALM and 25% less PLM neuronal defects at day 5, and 19% less ALM and 20% less PLM neuronal defects at day 10 as compared to DMSO‐treated *ucp‐4* mutants (Figure 4a–d). The decreased neuronal defects in DNP‐treated *ucp‐4* mutants are in agreement with the results of previous studies (Cho et al., 2016, 2017). At day 5, *ucp‐4* mutants treated with all of the tested flavonoids exhibited decreased ALM neuronal defects as compared to DMSO control (*p* < .05, Table S3 for *p*‐values, Figure 4a); at day 10, ALM neuronal defects were also decreased in *ucp‐4* mutants treated with all flavonoids except catechin (Figure 4c). In contrast, all tested flavonoids, except catechin, decreased PLM neuronal defects in *ucp‐4* mutants at both day 5 and 10 (Figure 4b and d). Quercetin showed the largest reducing effects in ALM neuronal defects at days 5 and 10; moreover, it also substantially reduced PLM neuronal defects at days 5 and 10. Chrysin at day 5 and naringenin at day 10 yielded the greatest reduction in PLM neuronal defects (26% and 28% reduction, respectively, Figure 4b and d). These results suggest that flavonoids mitigate neurodegeneration in aged *ucp‐4* mutants via mitochondrial uncoupling.

### 3.4 Flavonoids have no significant effects on ALM and PLM neuronal defects in *pink‐1;pdr‐1* double mutants

**Figure 5:**
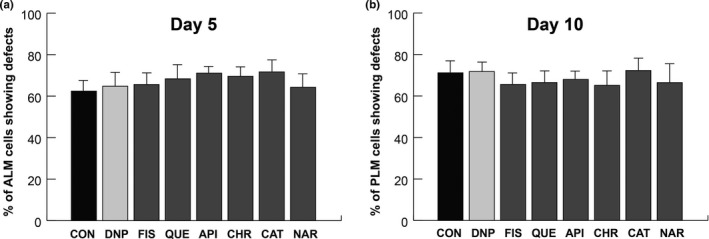
Flavonoids do not alleviate neurodegeneration in aged *pink‐1;pdr‐1* double mutants. (a and b). ALM and PLM neuronal defects are presented as a percentage of the total ALM‐ and PLM‐scored neurons in DMSO‐treated and flavonoid‐treated *pink‐1;pdr‐1* double mutants on day 5. DNP, 2,4‐dinitrophenol; FIS, fisetin; QUE, quercetin; API, apigenin; CHR, chrysin; CAT, catechin; NAR, naringenin; ALM, anterior lateral microtubule; PLM, posterior lateral microtubule. Data are represented as means of 3 independent experiments ± *SD*. *n* > 120 worms per line in each experiment. Chi‐squared tests were performed to compare flavonoids treatment and DMSO treatment. All *p*‐values > .05 (Table S4 for *p*‐values)

Recent studies have reported that mitophagy is the central process in mitochondrial quality control that influences the development of various diseases, including neurodegenerative diseases (Pickrell & Youle, 2015). The PINK1/Parkin pathway is a well‐known mitophagy process which has been evolutionarily conserved from simple animals such as *C. elegans* to much more complex animals like *Homo sapiens* (Palikaras et al., 2017). In addition, dissipation of mitochondrial membrane potential triggers mitophagy via PINK1 (Matsuda et al., 2010). Since the *pink‐1* single mutation can be rescued by overexpression of Parkin (Park et al., 2006), we examined the mechanosensory neuronal defects in both *pink‐1* and *pdr‐1* (*parkin* homolog) single mutants. Both *pink‐1* and *pdr‐1* mutants showed no significant difference in neuronal defects with flavonoid treatments and DMSO treatments (data not shown). Therefore, we used the *pink‐1;pdr‐1* double mutant in the *zdIs5* background to investigate whether mitophagy was involved in flavonoid‐mediated alleviation of neurodegeneration in aged *C. elegans*. First, we confirmed that there was no significant difference in the number of ALM and PLM defects between *pink‐1;pdr‐1* double mutants and *zdIs5* control worms at days 1–15 (data not shown). No significant differences in ALM and PLM neuronal defects were observed between flavonoid‐treated and DMSO‐treated worms at day 5 (Table S4 for *p*‐values, Figure 5a and b). Furthermore, neuronal defects at day 10 were similar to those at day 5 (data not shown). However, the possibility that flavonoids reduce neuronal defects in *pink‐1;pdr‐1* mutants due to their strong antioxidant activity cannot be ruled out.

The objective of this study was to investigate the mechanism by which flavonoids function as mild mitochondrial uncouplers to mitigate neurodegeneration in aged *C. elegans*. In this study, all flavonoid‐treated worms except catechin exhibited a reduction in neuronal defects and TMRE‐staining intensity despite the different physicochemical properties of various flavonoids. As expected, catechin induced slight reduction of both neuronal defects and TMRE intensities in accordance with weak uncoupling physicochemical properties of catechin, relatively high pKa = 9 (Figure 1). Therefore, the alleviation of neurodegeneration by flavonoids is likely due to common properties of flavonoids rather than to specific properties of each flavonoid. In addition, the favorable pKa and LogP values of flavonoids support our results (Stevens et al., 2018). Consistently, alleviation of neurodegeneration via mitochondrial uncoupling was achieved by treating aged *C. elegans* with the chemical uncoupler DNP in the present study as well as in previous studies (Cho et al., 2016, 2017). Moreover, our earlier studies demonstrated that neuronal defects were reduced in DNP‐treated uncoupling‐defective *ucp‐4* mutants (Cho et al., 2016, 2017). Taken together, these results indicate that flavonoids function as a mild mitochondrial uncoupler to alleviate neurodegeneration in aged *C. elegans*.

This study is significant in that the results show that (1) flavonoids act as mild mitochondrial uncouplers to dissipate mitochondria membrane potential, and (2) by doing so, flavonoids reduce neurodegeneration in aged *C. elegans*. It has been reported that PINK1 regulates mitochondrial integrity, particularly mitochondrial membrane potentials (Kondapalli et al., 2012). Therefore, the insignificant neuronal effects by flavonoids on *pink‐1;pdr‐1* mutants suggest a positive relationship between mitochondrial uncoupling by flavonoids and mitophagy in aging‐associated neurodegeneration (Kondapalli et al., 2012). However, further research on the relationship between flavonoid‐induced uncoupling and mitophagy in neurodegeneration must be done to obtain a comprehensive understanding of the mechanisms involved in the flavonoid‐mediated alleviation of aging‐associated neurodegeneration.

## CONFLICT OF INTEREST

The authors declare no conflict of interest.

## ETHICAL STATEMENT

This study does not involve any human or animal testing.

## Supporting information

Table S1‐S4
