# Supplementary material for: Flavonoids mitigate neurodegeneration in aged Caenorhabditis elegans by mitochondrial uncoupling
Source: Food Sci Nutr. 2020 Oct 23;8(12):6633–42. doi: 10.1002/fsn3.1956 (PMC7723185; doi:10.1002/fsn3.1956)
Supplement: Supplementary file 1 — Table S1‐S4 [file FSN3-8-6633-s001.docx]

Supplement Table 1. P values for N2 worms with flavonoids treatments. *P>0.05.

| EXPERIMENT | #1 |  |  |  |
| --- | --- | --- | --- | --- |
|  | DAY5 |  | DAY10 |  |
|  | ALM | PLM | ALM | PLM |
| CONTROL (N2) |  |  |  |  |
| DNP | 0.02000 | 0.03916 | 0.04007 | 0.00312 |
| FIS | 0.02486 | 0.03876 | 0.01238 | 0.00986 |
| QUE | 0.03669 | 0.00982 | 0.00267 | 0.01068 |
| API | 0.03669 | 0.01013 | 0.00470 | 0.00585 |
| CHR | 0.04455 | 0.00393 | 0.00513 | 0.00174 |
| CAT | 0.15315* | 0.00132 | 0.43732* | 0.00113 |
| NAR | 0.03465 | 0.00010 | 0.03433 | 0.00077 |
|  |  |  |  |  |
|  |  |  |  |  |
| EXPERIMENT | #2 |  |  |  |
|  | DAY5 |  | DAY10 |  |
|  | ALM | PLM | ALM | PLM |
| CONTROL (N2) |  |  |  |  |
| DNP | 0.01500 | 0.04708 | 0.04200 | 0.01775 |
| FIS | 0.04089 | 0.00515 | 0.01085 | 0.01170 |
| QUE | 0.00157 | 0.00621 | 0.04200 | 0.02862 |
| API | 0.03935 | 0.00061 | 0.04868 | 0.00472 |
| CHR | 0.04515 | 0.03019 | 0.04080 | 0.04779 |
| CAT | 0.22906* | 0.01303 | 0.11502* | 0.04966 |
| NAR | 0.04846 | 0.00075 | 0.03057 | 0.00136 |
|  |  |  |  |  |
|  |  |  |  |  |
| EXPERIMENT | #3 |  |  |  |
|  | DAY5 |  | DAY10 |  |
|  | ALM | PLM | ALM | PLM |
| CONTROL (N2) |  |  |  |  |
| DNP | 0.03100 | 0.00571 | 0.04426 | 0.02741 |
| FIS | 0.01708 | 0.02391 | 0.00775 | 0.00088 |
| QUE | 0.01023 | 0.00064 | 0.00032 | 0.00320 |
| API | 0.03572 | 0.01205 | 0.00449 | 0.04100 |
| CHR | 0.02252 | 0.02809 | 0.04269 | 0.00141 |
| CAT | 0.11091* | 0.02031 | 0.00551 | 0.00548 |
| NAR | 0.05109 | 0.02029 | 0.05051 | 0.01536 |

Table 2. P values for TMRE-stained N2 and ucp-4 worms with flavonoids treatments. *P>0.05.

| EXPERIMENT | #1 |  |  |
| --- | --- | --- | --- |
| CONTROL | N2 |  | *ucp-4* |
| DNP | 0.00441 |  | 0.00638 |
| FIS | 1.77E-09 |  | 2.91E-06 |
| QUE | 1.12E-09 |  | 9.71E-07 |
| API | 3.99E-06 |  | 8.95E-07 |
| CHR | 4.98E-07 |  | 6.63E-05 |
| CAT | 0.00545* |  | 0.01201 |
| NAR | 1.01E-07 |  | 0.00626 |
|  |  |  |  |
|  |  |  |  |
| EXPERIMENT | #2 |  |  |
| CONTROL | N2 |  | *ucp-4* |
| DNP | 0.01616 |  | 0.00111 |
| FIS | 1.53E-05 |  | 7.63E-07 |
| QUE | 5.36E-06 |  | 9.87E-11 |
| API | 0.00012 |  | 1.06E-07 |
| CHR | 0.00018 |  | 7E-08 |
| CAT | 0.17019* |  | 0.00278 |
| NAR | 0.00039 |  | 1.08E-06 |
|  |  |  |  |
|  |  |  |  |
| EXPERIMENT | #3 |  |  |
| CONTROL | N2 |  | *ucp-4* |
| DNP | 9.54E-05 |  | 2.94E-05 |
| FIS | 6.96E-08 |  | 2.88E-08 |
| QUE | 3.5E-07 |  | 3.75E-09 |
| API | 9.6E-06 |  | 1.98E-06 |
| CHR | 3.58E-06 |  | 1.08E-07 |
| CAT | 0.00026 |  | 0.00049 |
| NAR | 1.82E-09 |  | 0.00014 |

Table 3. P values for *ucp-4* worms with flavonoids treatments. *P>0.05.

| EXPERIMENT | #1 |  |  |  |
| --- | --- | --- | --- | --- |
|  | DAY5 |  | DAY10 |  |
|  | ALM | PLM | ALM | PLM |
|  |  |  |  |  |
| DNP | 0.00009 | 0.00023 | 0.00024 | 0.00105 |
| FIS | 0.00270 | 0.00036 | 0.00077 | 0.00005 |
| QUE | 0.00011 | 0.00034 | 5.34E-07 | 0.00002 |
| API | 0.01716 | 0.00474 | 0.00213 | 0.00026 |
| CHR | 1.48E-06 | 0.00081 | 0.00134 | 0.00190 |
| CAT | 0.00024 | 0.70806* | 0.00015 | 0.05390* |
| NAR | 0.00071 | 0.02862 | 0.00019 | 2.83E-06 |
|  |  |  |  |  |
|  |  |  |  |  |
| EXPERIMENT | #2 |  |  |  |
|  | DAY5 |  | DAY10 |  |
|  | ALM | PLM | ALM | PLM |
|  |  |  |  |  |
| DNP | 0.00692 | 0.00033 | 0.00072 | 0.00007 |
| FIS | 0.00355 | 0.00017 | 0.01074 | 6.18E-08 |
| QUE | 0.00017 | 0.00059 | 5.16E-07 | 2.35E-07 |
| API | 0.01314 | 0.00405 | 0.03537 | 0.00032 |
| CHR | 0.00086 | 0.00007 | 0.00116 | 0.00007 |
| CAT | 0.01893 | 0.34257* | 0.55967* | 0.03429 |
| NAR | 0.03638 | 0.01017 | 0.00081 | 3.77E-07 |
|  |  |  |  |  |
|  |  |  |  |  |
| EXPERIMENT | #3 |  |  |  |
|  | DAY5 |  | DAY10 |  |
|  | ALM | PLM | ALM | PLM |
|  |  |  |  |  |
| DNP | 0.00018 | 0.00108 | 0.00085 | 0.00027 |
| FIS | 0.00077 | 0.00259 | 0.01158 | 0.00004 |
| QUE | 0.00005 | 0.00145 | 0.00015 | 1.92E-06 |
| API | 0.00973 | 0.01863 | 0.00278 | 0.00051 |
| CHR | 0.00126 | 0.00037 | 0.02879 | 0.00014 |
| CAT | 0.00191 | 0.72328* | 0.12147* | 0.05835* |
| NAR | 0.02220 | 0.03538 | 0.00003 | 0.00000 |

Table 4. P values for *pink-1;pdr-1* worms with flavonoids treatments.

| EXPERIMENT | #1 |  |
| --- | --- | --- |
|  | DAY5 |  |
|  | ALM | PLM |
| *pink-1;pdr-1* |  |  |
| DNP | 0.32520 | 0.17479 |
| FIS | 0.13638 | 0.14342 |
| QUE | 0.24069 | 0.03409 |
| API | 0.09450 | 0.09460 |
| CHR | 0.15113 | 0.14651 |
| CAT | 0.03580 | 0.76930 |
| NAR | 0.28114 | 0.24532 |
|  |  |  |
|  |  |  |
| EXPERIMENT | #2 |  |
|  | DAY5 |  |
|  | ALM | PLM |
| *pink-1;pdr-1* |  |  |
| DNP | 0.88879 | 0.61820 |
| FIS | 0.53116 | 0.76034 |
| QUE | 0.23145 | 0.83654 |
| API | 0.96255 | 0.91860 |
| CHR | 0.34389 | 0.79234 |
| CAT | 0.16589 | 0.38266 |
| NAR | 0.99825 | 0.72510 |
|  |  |  |
|  |  |  |
| EXPERIMENT | #3 |  |
|  | DAY5 |  |
|  | ALM | PLM |
| *pink-1;pdr-1* |  |  |
| DNP | 0.95500 | 0.22347 |
| FIS | 0.33590 | 0.26616 |
| QUE | 0.48085 | 0.44358 |
| API | 0.00826 | 0.79213 |
| CHR | 0.19403 | 0.11988 |
| CAT | 0.16755 | 0.86276 |
| NAR | 0.83836 | 0.10277 |
